# OA02.03. Nutrient biomarker patterns and rates of cognitive decline in dementia-free elders

**DOI:** 10.1186/1472-6882-12-S1-O7

**Published:** 2012-06-12

**Authors:** G Bowman, J Quinn, J Kaye, J Shannon

**Affiliations:** 1 Oregon Health & Science University, Portland, USA

## Purpose

We previously identified three distinct nutrient biomarker patterns associated with both psychometric and neuroimaging indices of brain health in a cross-sectional analysis. The objective of this study was to examine the relationship between the nutrient biomarker patterns and cognitive decline over 2 years.

## Methods

Thirty biological markers of diet were assayed in plasma from 104 dementia-free elders in the Oregon Brain Aging Study. Principal component analysis constructed distinct nutrient biomarker patterns. A linear regression model was used to assess the association between NBPs and rate of change in Clinical Dementia Rating - sum of box score over two years.

## Results

Mean age was 87±10, 62% were female, and 10% were carrying the ApoEe4 allele. Two distinct nutrient biomarker patterns were associated with rates of cognitive decline: a vitamin pattern high in B, C, E and D (p=0.001) and a high trans fat pattern (p<0.001) associated with less and more decline over two years, respectively. These findings were independent of age, gender, education years, ApoEe4 carrier status and vascular risk factors.

## Conclusion

A plasma nutrient profile high in certain vitamins and low in trans-fat may be prudent for maintaining cognitive function in older populations.

